# The nature, scope and impact of genomic prediction in beef cattle in the United States

**DOI:** 10.1186/1297-9686-43-17

**Published:** 2011-05-15

**Authors:** Dorian J Garrick

**Affiliations:** 1 Department of Animal Science, Iowa State University, Ames, IA 50011-3150, USA; 2 Institute of Veterinary, Animal & Biomedical Sciences, Massey University, Palmerston North, New Zealand

## Abstract

Artificial selection has proven to be effective at altering the performance of animal production systems. Nevertheless, selection based on assessment of the genetic superiority of candidates is suboptimal as a result of errors in the prediction of genetic merit. Conventional breeding programs may extend phenotypic measurements on selection candidates to include correlated indicator traits, or delay selection decisions well beyond puberty so that phenotypic performance can be observed on progeny or other relatives. Extending the generation interval to increase the accuracy of selection reduces annual rates of gain compared to accurate selection and use of parents of the next generation at the immediate time they reach breeding age. Genomic prediction aims at reducing prediction errors at breeding age by exploiting information on the transmission of chromosome fragments from parents to selection candidates, in conjunction with knowledge on the value of every chromosome fragment. For genomic prediction to influence beef cattle breeding programs and the rate or cost of genetic gains, training analyses must be undertaken, and genomic prediction tools made available for breeders and other industry stakeholders. This paper reviews the nature or kind of studies currently underway, the scope or extent of some of those studies, and comments on the likely predictive value of genomic information for beef cattle improvement.

## Background

Genetic improvement results from selection of above-average candidates as parents of the next generation. In a competitive market, above-average candidates would be those that improve consumer satisfaction, influencing immediate eating quality, purchase cost, long-term health implications of consumption, care of the environment in the production and processing of the beef; and welfare of the animals. Satisfied consumers demand and pay more for desirable beef, and under perfect competition this will be reflected along the production chain by increased farm-gate prices for cow-calf producers. Seedstock suppliers that sell bulls to cow-calf producers would be expected to respond by developing and implementing breeding programs that provide successive crops of bulls that outperform their predecessors.

Inspection of genetic trends, e.g. [1,2], shows that beef cattle selection has resulted in animals with increased merit for early growth and improved rib eye area and marbling scores. There is no evidence for genetic improvement in reproductive performance. Selection has resulted in animals with larger mature size [1] and greater cow maintenance requirements [2], which increase production costs, as cow maintenance requirements are a major determinant of the total feed required in the production system [3]. Beef cattle selection has therefore failed in practice to achieve balanced improvement across the spectrum of traits that contribute to breeding goals. One reason has been our inability to cost-effectively rank selection candidates for all the attributes of interest [4]. This is the case because reliably quantifying the merits of animals in terms of their breeding values has been totally reliant on recording pedigree and performance information, primarily on the selection candidates themselves, their parents and perhaps their offspring. This has led to improvement programs that have been phenotype driven, i.e. programs that are focused on easy to measure traits that are recorded at young ages, such as early growth and ultrasound assessment of carcass attributes, rather than being goal driven and focused on all the attributes that influence consumer satisfaction [5]. The fundamental reason for this failure is that mixed model predictions of merit using the relationship matrix and applied to young animals can, with sufficient historical data, reliably predict the parent average (PA) effects, but are unable to predict the Mendelian sampling effects without having phenotypic observations on the individual or its descendants [6]. Accordingly, with only ancestral records, there is little information to discriminate among paternal half-sibs other than based on the merit of the dams. In that setting, it is seldom possible to identify young selection candidates with merit superior to existing selected sires. In the beef cattle context, this has led to low selection accuracy for mature size, lifetime reproductive performance, stayability/longevity, and disease resistance. Other important traits such as tenderness of beef, other aspects of eating quality, and feed efficiency, have had no prospects for selection as there are no phenotypic measures that can be readily and cost-effectively obtained on large numbers of seedstock animals.

Molecular-based information has long held promise to improve the prediction of young animals by first using phenotypic markers, second using microsatellite markers, and most recently using ever-increasing densities of single nucleotide polymorphisms (SNP).

Phenotypic markers such as blood groups were found to characterize the inheritance of certain chromosomal regions, proving useful for selection if that region contained a major gene responsible for variation in a trait of interest [7]. Unfortunately, there are insufficient simply inherited phenotypic attributes to characterize the entire genome.

Highly polymorphic microsatellite markers provided new opportunities to find major genes or quantitative trait loci (QTL) that influence important traits [8]. These markers that can have many alleles at each locus, can be informative in much of the population, and are well distributed along the genome. The offspring of any heterozygous parent can be segregated on the basis of marker information, to distinguish the marker haplotype inherited from each parent in a particular genomic region. Microsatellite genotyping was and is expensive and consequently many experiments lacked sufficient power to characterize regions well, and therefore detected only the largest effects [9]. Relatively few QTL were found that were useful for beef cattle improvement [10], although many interesting scientific discoveries arose from these endeavors.

Following the sequencing of the bovine genome, which led to the discovery of millions of bi-allelic SNP, and the creation of subsets of SNP that can characterize the genome and be multiplexed for cheap and efficient genotyping [11], molecular-based studies to predict animal merit have been based on high-density SNP genotypes. This review documents the current status of whole-genome prediction of breeding merit in beef cattle and describes its implementation for the purposes of selection.

### Breeding objective

The breeding objective comprises a list of traits that influence the breeding goal, along with their relative emphasis [12]. An ideal breeding objective would include all the traits that will in the future influence the breeding goal. A profit-based goal would motivate the list to include all attributes that will influence income or costs. For beef cattle, these clearly include: traits that influence productivity such as reproductive performance, growth rate and survival; traits that influence cost of production such as feed intake; and traits that influence product quality such as tenderness and taste. In recent times, the list of traits has been expanding to include attributes that have been externalities. These include traits that impact the long-term contribution of beef consumption on human healthfulness, such as factors that influence anemia, cancer, obesity, diabetes and heart disease; traits that influence the environment in its broadest context, comprising air quality, water quality, soil degradation, visual farm/feedlot appearance and competition with wildlife throughout the production, finishing and processing system; and welfare factors, both of the animals in terms of exhibiting natural behaviors and being free of disease, suffering, and mortality, and of the labor in terms of worker safety. In this context, the design of a beef cattle improvement program should holistically consider traits that influence production efficiency such as individual animal measures of inputs and outputs, traits that influence the quality of the eating experience, traits that influence animal health, and traits that influence the human healthfulness of the consumed beef.

The tools available to the animal breeder to improve consumer satisfaction from beef include: the choice of breed, the choice of mating plan to exploit complementarities and heterosis, and selection for within-breed improvement [12]. The main tools for selection for within-breed improvement are the estimated breeding values (EBV) and corresponding indexes that arise from national cattle evaluations (NCE), which are available in many countries and empowers genetic improvement within the seedstock sector [4]. In the absence of genotype-environment interactions that can occur when seedstock animals are managed in different and typically superior environments compared to those of commercial animals [13], those gains are passed on to the commercial cow-calf sector by the sale of improved bulls (or semen) to be used as sires.

The current focus of the use of genetic markers for genomic prediction is to improve within-breed selection, by increasing the accuracy of existing EBV by the time the selection candidate reaches puberty, or by providing new EBV for attributes that influence the breeding goal but have not been available from conventional performance recording. Other genomic analyses that will not be considered in this review include correct assignment of parents, identification of genetic diseases, detection of signatures of selection, prediction of breed composition of crossbred animals and identification of QTL.

### Estimated breeding values from national cattle evaluations in the United States

National cattle evaluations (NCE) in beef cattle began with measures of weight traits, and now include birth, weaning and yearling weights, and to a lesser extent mature weights. Rather than reporting EBV, US breed associations typically report Expected Progeny Differences (EPD), that are one-half the EBV. A summary of the traits for which EPD are typically reported is in Table 1 for the 16 most prominent US beef cattle breeds. Calving ease has been added to most national evaluation systems and, like weaning weight, includes EPD that reflect direct and maternal contributions [14]. Carcass traits have typically been problematic to collect in seedstock herds, so most carcass information tends to come from ultrasound measures of rib-eye area (REA), intramuscular fat (IMF) and fat depth [4]. Not all breed associations provide carcass EPD. Eating quality is principally limited to tenderness, but this is difficult to measure in most processing plants. In the US, carcass marbling has been used as a surrogate for tenderness/eating quality. More recently, QTL in the region of the calpain and calpastatin genes have been exploited for marker-assisted selection, using SNP that vary among breeds, most notably between *Bos indicus *and *Bos taurus *breeds. Reproductive measures have been difficult to evaluate since most breed associations have not used inventory recording systems until relatively recently, so it is impossible to determine if a female not represented as a dam actually calved or not [5]. Reproductive EPD have therefore been limited to scrotal circumference, and more recently, heifer pregnancy. There are no routine measures of input traits on a significant scale, as feed intake is problematic to measure, especially in grazing circumstances. Maintenance energy requirements have been predicted from knowledge on mature weight, condition score and milk production potential [3].

**Table 1:** Traits reported in national cattle evaluation for the 16 most prominent beef cattle breeds in the US

Biotype	British				Continental							Indicus (and cross)				
**Breed**^**2**^	**AAA**	**AHA**	**RAA**	**ASH**	**AIC**	**AGA**	**AMA**	**ASA**	**BAA**	**NAL**	**SAL**	**ABB**	**ACA**	**BBU**	**IBB**	**SGA**
**Trait**^**1**^																

BWT	×	×	×	×	×	×	×	×	×	×	×	×	×	×	×	×
WWT	×	×	×	×	×	×	×	×	×	×	×	×	×	×	×	×
Milk	×	×	×	×	×	×	×	×	×	×	×	×	×	×	×	×
YWT	×	×	×	×	×	×	×	×	×	×	×	×	×	×	×	×
YHT	×															
MWT	×															
MHT	×															
CCW	×		×	×	×	×	×	×	×	×	×	×	×			×
MRB	×	×	×	×	×	×	×	×	×	×		×	×	×	×	×
REA	×	×	×	×	×	×	×	×	×	×	×	×	×	×	×	×
FAT	×	×	×	×	×		×	×			×	×	×	×	×	×
RUMP														×		
YLD			×	×			×	×		×	×	×	×			
WBSF								×				×				
CED	×	×	×	×	×	×		×	×	×	×					
CEM	×	×	×	×	×	×		×	×	×						
SC	×	×			×	×				×	×			×	×	
HPG			×													
STAY			×			×		×								
GL						×										
DOC	×									×	×					
RADG	×															
ME			×													
DTF						×										

Trait^1^: BWT = birth weight, WWT = weaning weight direct, Milk = weaning weight maternal, YWT = yearling weight, YHT = yearling height, MWT = mature weight, MHT = mature height, CCW = carcass weight, MRB = marbling/intramuscular fat, REA = rib eye area, FAT = fat depth (usually over rib), RUMP = fat depth over rump, YLD = retail beef yield/percent retail cuts/yield grade, WBSF = Warner-Bratzler shear force (tenderness), CED = calving ease direct, CEM = calving ease maternal, SC = scrotal circumference, HPG = heifer pregnancy rate, STAY = stayability, GL = gestation length, DOC = docility, RADG = residual average daily gain, ME = maintenance energy requirements, DTF = days to finish

Breed^2^:British: AAA = American Angus Association, AHA = American Hereford Association, RAA = Red Angus Association of America, ASH = American Shorthorn Association; Continental: AIC = American International Charolais Association, AGA = American Gelbvieh Association, ASA = American Simmental Association, BAA = Braunvieh Association of America, AMA = American Maine Anjou Association, NAL = North American Limousin Foundation, SAL = American Salers Association; Indicus: ABB = American Brahman Breeders Association, ACA = American Chianina Association (includes Chiangus), BBU = Beefmaster Breeders United, IBB = International Brangus Breeders Association, SGA = Santa Gertrudis Association

### Genomic prediction

The concept of using high-density SNP genotypes to predict genetic merit was popularized by the landmark publication of Meuwissen *et al*. [15]. Their approach involved the computation of EBV for individual chromosome fragments, characterized by SNP genotypes or haplotypes. Estimated breeding values of selection candidates are subsequently obtained by summing up the values of all inherited chromosome fragments. This estimate is referred to as a molecular breeding value (MBV). A variety of methods has been proposed to derive EBV of chromosome fragments [16], and these can be broadly categorized into methods that fit all SNP, and methods that use mixture models that assume that not all but a fraction of the SNP have effects on the trait. All methods can be reparameterized in terms of equations that fit animal genetic effects rather than SNP effects and obtain the MBV directly, using the inverse of a genomic-based rather than a pedigree-based relationship matrix in the mixed model equations [17]. The concept of genomic prediction using a genotype-based relationship matrix predates [15] by several years [18]. In practice, so-called genomic training populations that are used to derive prediction equations, may be of inadequate size for reliable prediction of all but the largest chromosome fragments [19], leading to predictions that account for just a fraction of the additive genetic variance [20]. In this circumstance, blending the MBV and the conventional PA will improve accuracy [21]. Given the genotypes, blending can be achieved in the same analysis as the genomic training, using an inverse relationship matrix constructed from pedigree information on non-genotyped individuals and genomic information on genotyped animals [22,23]. In the absence of the genotypes, the blending can be achieved using MBV as a correlated trait [24]. That approach requires knowledge of the covariance components relating the MBV to the trait, typically represented in publications as the genetic correlation [25,26].

Whereas microsatellite marker studies have typically failed to identify QTL and subsequently SNP that could apply equally well across a range of breeds, there was hope that the reduced cost and the increased density of multiplexed SNP panels would lead to discoveries that could be exploited across breeds. The reduced cost per genotype for panels of 50,000 or more multiplexed SNP compared to microsatellite markers allows for more animals to be used in analyses, increasing power. In both conventional QTL studies and in genomic prediction, detection of effects relies on an association between the segregating marker genotype and the segregating causal polymorphism. The strength of this association reflects the extent of linkage disequilibrium (LD), which can be represented by the squared correlation between genotypes at two loci. Microsatellite studies exploited linkage relationships to create LD between the flanking sparse markers and a QTL within families, even when the marker was in linkage equilibrium with the QTL from a population perspective. Genomic prediction does not require family structures but takes advantage of the higher density of SNP markers and the fact that physically close loci tend to have higher LD than distant loci. Provided the genome is saturated with SNP markers, any QTL should be near some genotyped SNP and hopefully at least one will be in sufficient LD with the QTL.

Research studies of genomic prediction in livestock populations began with the release by Illumina of a high-density bovine panel of some 54,001 SNP markers [27]. In any particular breed, a proportion of these SNP will not be segregating, so the genotypes will be described in this paper as coming from a 50k panel.

### Beef cattle training populations

Training involves statistical analyses that exploit individuals with both high-density genotypes and recorded performance [28]. The amount of data required for training depends upon a number of factors, including the heritability of the trait [29]. One approach to training is to use sires whose genetic merit can be assessed more reliably using progeny performance than would be the case using only measurements on the individual sire itself [9]. This may be more problematic in beef cattle than dairy cattle, as the recorded population of even the largest beef cattle breed is much smaller than that of the Holstein breed. Further, artificial insemination (AI) is much less used in beef cattle seedstock herds than in dairy herds, collectively resulting in fewer highly reliable sires available for use in training.

Industry populations have advantages for genomic prediction. In the case of elite or widely used industry animals, the individuals included in the training data will be relevant to the commercial population. For AI sires, DNA is readily accessible despite the disparate ownership or physical location of the animals. The principal source of performance information comes as EPD from NCE and is well represented for growth traits, moderately well for ultrasound traits, poorly for behavior, reproduction and longevity traits, and typically with no information on many other traits such as disease resistance or eating quality. Since most recorded animals are purebred, training on crossbred data is seldom an option using NCE data and is limited to those few breed associations that collect crossbred data.

A US repository of DNA from over 3,000 Angus bulls born since 1948 was assembled by the University of Missouri [30]. These bulls are represented in American Angus Association pedigrees and have generally been widely used. Accordingly, these bulls have EPD and accuracies for *production traits*: calving ease (direct); birth weight; weaning weight; yearling weight; yearling height; scrotal circumference; *maternal traits*: maternal calving ease; milk; mature weight; mature height; *carcass traits*: carcass weight; marbling; rib eye area; fat depth; along with some *newly released *trait EPD: docility; and heifer pregnancy. The accuracies of EPD on old bulls are limited for some traits. Igenity, a genomic testing service owned by the animal health company Merial, has used the results from the analysis of this Angus population, along with other resource populations, to market a reduced panel comprised of a subset of informative SNP referred to as a 50k-derived product. It is marketed in the US in conjunction with the American Angus Association and costs $65 [31].

The US Meat Animal Research Center (US-MARC) at Clay Center Nebraska has worked with some breed associations to develop a repository of some 2,026 influential or upcoming bulls in 16 of the most prominent beef breeds in the US with EPD from NCE and includes: Angus, Beefmaster, Brahman, Brangus, Braunvieh, Charolais, Chiangus, Gelbvieh, Hereford, Limousin, Maine-Anjou, Red Angus, Salers, Santa Gertrudis, Shorthorn, and Simmental. Initial plans for the use of this repository were to provide genomic predictions of these bulls from training analyses based on a US-MARC crossbred population [32] and to carry out multi-breed training. These SNP genotypes have now been made available to the respective breed associations.

The alternative to training on widely-used sires is to train using phenotypes collected specifically for genomic analyses. This could be achieved using non seedstock field data, but in many cases the mating designs and contemporary group classifications are not entirely adequate for the purpose. Most field data comprise offspring from natural mating, so sires tend to be nested within rather than cross-classified by contemporary groups. In the case of carcass traits, animals tend to have their ownership transferred several times between weaning and harvest, making it difficult to ensure harvest cohorts were managed together throughout their entire lifetime. For reproductive traits, it is difficult to obtain sizeable cohorts of animals for comparison, particularly for phenotypic measurements obtained after first calving, as birth cohorts get subdivided according to sex of calf, age of dam, and whether or not yearlings became pregnant. These problems can be overcome by sourcing animals from large herds and by designing the study prior to the birth of the study animals, which may be several years prior to the collection of phenotypes.

The US carcass merit project (CMP) was one such long-term industry-funded semi-structured undertaking initiated in 1998 that collected carcass data, tenderness and sensory attributes on over 8,200 progeny. Some of the half-sib offspring of more than 70 sires across 13 breeds were DNA sampled. The sires were widely-used AI bulls from various breeds and dams were commercial cows [33]. The dataset has been valuable to validate early genomic tests being commercialized in the US. Validation of tests using these data has been undertaken by the National Beef Cattle Evaluation Consortium (NBCEC) and the details having been published on-line by Van Eenennaam et al. [34]. More recently, the CMP dataset has been genotyped using high-density SNP chips by at least two different organizations to identify genes and to apply whole-genome prediction, which will prevent this resource from being used for independent validation of future tests derived from that data.

Collecting data for more novel phenotypes requires the deliberate generation of suitable populations. Given the current dominant market position of the Angus breed in the US, it was an obvious candidate for any new studies to expand the scope of traits for genomic prediction. Two large studies have been undertaken, one at Iowa State University to investigate fatty acid and mineral content in beef as possible targets for improving the human healthfulness of beef, and another at Colorado State University to investigate feedlot health. The healthfulness study involved several cohorts representing 2,300 predominately Angus cattle assessed for carcass and meat quality attributes, including tenderness and sensory information, in addition to extensive phenotyping of traits that might influence the human healthfulness of beef. These healthy beef traits include mineral and fatty acid compositions of key muscles [35]. The feedlot health study used two annual crops of about 1,500 composite British and Continental steers from one ranch in Nebraska. The animals were extensively phenotyped for feedlot health, particularly respiratory disease and response to treatment. Sickness was assessed visually, by temperature profiles and by lung damage scores. Data includes temperament and immunological measures [36]. Both experiments included body weight and a number of carcass and meat quality phenotypes. These collective resources have been used, along with other populations, to develop an Angus 50k product for production and carcass traits that Pfizer Animal Genetics has marketed in the US for $124-$139, depending upon the number of animals tested [37], with predictions from this panel now incorporated in NCE undertaken for the American Angus Association.

Research herds with deep phenotyping are also candidates for studies of genomic prediction. The most comprehensive such resource is represented by the US-MARC germplasm evaluation studies, the recent cohorts being known as the Cycle VII and F1-squared populations. In addition to an across-breed training analysis for which single-SNP effects have been published for birth, weaning and yearling weights and their respective gains [38], this population was used to develop a low-density 196-SNP panel with markers believed to be informative for weaning weight. Such reduced panels comprised of only the most informative markers were believed to be more cost-effective and therefore more likely to be widely adopted by the beef industry. That panel was used in a project coordinated by the NBCEC to demonstrate the use of reduced panels in seedstock herds, and the incorporation of the resulting MBV into NCE [39].

The collection of feed intake on large numbers of animals is still problematic from a practical viewpoint, and to date, such data has been limited to measuring relatively small disparate groups of animals during finishing, with findings focused on QTL detection rather than genomic prediction. Other datasets of limited size have been collected on a range of traits, including reproductive performance and tick resistance but have not yet had any findings published from a genomic prediction perspective.

### Funding for genotyping training populations

Costs for conventional pedigree and performance recording and for NCE have been met by producer funds in the US. Public funds have been used for the development of NCE methodology. Public funds were not immediately available for extensive genotyping of training populations, and neither seedstock breeders nor breed associations had funds to adopt this technology beforehand given the uncertain nature of its value. Fortunately, applications of this approach in beef cattle improvement were considered as business opportunities by commercial companies such as Merial Igenity and Pfizer Animal Genetics to invest in the training phase, presumably with expectations of recouping returns on that investment through future sale of genomic tests. However, this situation has changed industry dynamics, introducing competitive partners into the process of ranking animals, and has increased the proprietary nature of performance information, genotypes and analytical approaches. This is one reason for the dearth of refereed publications on the accuracy of genomic prediction in beef cattle, in contrast to the dairy cattle situation.

### Predictive ability of whole-genome findings

Confidence in genomic predictions can only be provided by validation in a group of animals that are not included in the training population. Close relationships between animals in training and validation populations tend to lead to better predictive ability than when the groups are more distantly related [40]. Analysis of simulated data suggests that methods based on mixture models provide better predictive ability than methods that assume all the SNP have predictive value [15], while analysis of field data tends to demonstrate relatively little difference between alternative methods, and some inconsistencies appear from trait to trait as to which is the most predictive method [41,42]. There appears to be more variation in predictive ability according to the choice of validation population than there is between methods.

### Within-breed 50k predictions

One of the few reports on accuracy of genomic predictions in beef cattle analysed deregressed EPD [43] from NCE to quantify cross-validation results from 2,100 Angus AI bulls [44]. The data were partitioned into three subsets, with training animals in two groups and validation animals in the third. Subsets were created so that no sire had sons in both the training and validation groups. Genomic predictions were obtained from the training data using method Bayes C [41]. Predictive ability was quantified as correlations between 50k predictions and realized (deregressed) performance (Table 2). The general conclusion is that correlations between genomic predictions from 50k SNP and deregressed EPD in independent datasets of related animals are 0.5-0.7. It is not possible from these correlations to readily derive the genetic correlation between genomic prediction and the true BV, because of heterogeneity of variance among the deregressed EPD. This heterogeneity does not impact the expectation of the estimated covariance between genomic predictions and deregressed EPD, but it does impact the estimated variance of the deregressed EPD. Furthermore, the genotyped animals represent AI sires, and these represent highly selected individuals, so their genetic variance is not likely to be representative of the population genetic variance. Also, correlations between genomic prediction and EPD do not provide expectation on the genetic correlation, due to the varying degrees of shrinkage influencing EPD, which vary in their information content. Accordingly, correlations between genomic prediction and EPD or deregressed EPD provide a guide to accuracy, but cannot be interpreted as quantifying the proportion of variation accounted for by the genomic prediction applied to new animals. This would not be the case for correlations between genomic prediction and homogeneous information such as individual phenotypic observations.

**Table 2:** Correlations of 50k or 600 SNP predictions with deregressed EPD for various traits using cross-validation with three subsets of the data

Trait	Training 2 and 3 Prediction 1 (50k)	Training 1 and 3 Prediction 2 (50k)	Training 1 and 2 Prediction 3 (50k)	**Overall**^**1 **^**(50k)**	Overall (600 SNP)
FAT	0.71	0.64	0.73	0.69	0.63
CED	0.65	0.47	0.65	0.59	0.61
CEM	0.58	0.56	0.62	0.53	0.55
MRB	0.72	0.73	0.64	0.70	0.67
REA	0.63	0.63	0.60	0.62	0.56
SC	0.60	0.57	0.50	0.55	0.51
WWD	0.65	0.44	0.66	0.52	0.49
YWT	0.69	0.51	0.72	0.56	0.55

Traits: backfat (FAT), calving ease direct (CED) and maternal (CEM), carcass marbling (MRB), ribeye area (REA), scrotal circumference (SC), weaning weight direct (WWD) and yearling weight (YWT); ^1^correlation estimated by pooling estimated variances and covariances

Other numerically important breeds tend to have fewer registrations than Angus and it will be difficult to collect comparable sized training populations of AI sires. In contrast to the dairy industry, most bulls are used solely in commercial herds that do not record parentage or individual performance and therefore do not obtain progeny information for training or validation. The American Hereford Association has increased the 50k genotypes provided by US-MARC to develop a training population of 800 animals, but no results have been published yet. The other US breeds have even fewer animals ready for training.

Genomic prediction for beef cattle healthfulness has shown varying levels of predictive ability, as determined by the proportion of variation accounted for by markers [35]. Using samples from the *Longissimus dorsi*, iron concentration of beef could be readily predicted, whereas magnesium, manganese, phosphorus and zinc concentrations appeared to be under less genetic control. For other minerals such as calcium, copper, potassium and sodium, concentrations could not be predicted. Prediction of the fatty acid's concentrations showed similar trends to that of the minerals' concentration. For the predominant even-numbered saturated fatty acids C14:0, C16:0 and C18:0, monounsaturated C18:1 and polyunsaturated C18:2, prediction was good, while for C18:3 and conjugated linoleic acid (CLA) concentrations, predictions were not conclusive. These results look promising to develop tools capable of modifying the concentration of saturated fatty acids, or the relative proportions of saturated and unsaturated fatty acids. For these traits, the challenge will consist in developing a market for beef with modified fatty acid composition.

Using the same dataset as for beef healthfulness, it has been shown that carcass and beef quality traits can be predicted [35]. Hot carcass weight, calculated yield grade, marbling score and fat thickness had 40-50% of phenotypic variance explained by the 50k markers, whereas markers accounted for less than 30% of the variation for dressing percentage, loin eye area and tenderness assessed by Warner-Bratzler shear force. Cross validation results were not reported.

### Within-breed reduced panels

Reduced SNP panels can be produced either to be highly informative for a particular trait or for several traits by including the most strongly associated SNP, or to be informative for high-density genomic prediction after imputing the high-density panel from a reduced set of evenly spaced SNP with high minor allele frequency [45]. To date, the beef industry focus has been on subsets of markers chosen to be informative for a subset of traits that are believed to have the most economic relevance and greatest market opportunity.

Mixture models such as Bayes B and Bayes C [41] assume that some fraction of the SNP have zero effect on the trait. The posterior frequency with which any particular SNP was fitted in an MCMC analysis reflects the informativeness of particular SNP and can be used for SNP selection. Subsets of 600 SNP markers created by selecting the 20 markers on each bovine chromosome with the highest model frequency, from Bayes C analyses with 90% of 50k SNP assumed to have zero effect, demonstrated relatively little loss of predictive ability compared to 50k predictions [43]. Cheaper genotyping can be achieved by reducing the number of markers to a single set of 384 SNP, chosen for predictive ability across the portfolio of traits of interest. However, reducing the number of SNP below 600 reduces predictive ability. For example, the correlation reported in [43] for sets of the best 50, 100, 150 or 200 SNP chosen to predict marbling in Angus were 0.28, 0.29, 0.39, and 0.43, well below the 0.67 achieved with 600 SNP. A single set of 384 markers chosen from the above analysis for predictive ability across a range of traits was validated in a new population of 275 Angus bulls [43]. The correlations from that analysis were 0.59 for marbling, 0.32 for backfat, 0.58 for rib eye, 0.44 for carcass weight, 0.39 for heifer pregnancy and 0.35 for yearling weight.

In the study on beef healthfulness [35], subsets of as few as 10 markers retained more than half of the predictive ability of the 50k SNP chip when used to predict the even-numbered saturated fatty acids C14:0 and C16:0. The genomic architecture of mineral and fatty acid concentrations is likely to be much simpler, as the biochemical pathways and enzymes involved in metabolizing and catabolizing these compounds have been identified and seem to be somewhat straightforward, in contrast to traits such as growth rate, which are the collective result of genes influencing bone growth, muscle growth, fat accumulation, visceral weight among other factors.

The development of reduced panels for any quantitative trait in breeds other than Angus is currently limited by the lack of training populations. In contrast to the dairy industry, where reduced panels are being used for imputation of 50k markers for genomic prediction [46], target populations in beef cattle are diverse in terms of species (*Bos indicus *and *Bos taurus*) and breeds. Furthermore, many pre-pubertal selection candidates are offspring of natural mating rather than of AI sires. Collectively, these facts increase the genetic distance between the training and target populations.

### Across-breed panels

Prediction across breeds is more problematic because different breeds may exhibit different QTL, dominance or epistasis can occur, and allele frequencies may vary between populations. Linkage disequilibrium (LD) is not very consistent across breeds and therefore training in one beef cattle breed using 50k genotypes will not be very effective to predict a different breed [47]. Simulated data using actual 50k genotypes from the CMP and an Angus dataset as if they were causal genes and adding a random environmental effect to represent a trait with 50% heritability, demonstrated that predictive ability varied according to the number of simulated QTL. The best results were achieved for the smallest number of QTL, since in that scenario the average size of the QTL was larger than when more QTL were simulated. The across-breed predicted correlation from the simulation [47] varied from a high of 0.4 for 50 QTL down to 0.2-0.3 for 500 QTL. These correlations account for up to 18% of genetic variance for 50 genes and less than 10% of variance for 500 genes. Unpublished data predicting the merit of Hereford bulls using training results from Angus bulls always resulted in positive correlations, but typically less than 0.10, with the best correlation being 0.18 for birth weight and slightly less for yearling weight. Genomic prediction in beef cattle based only on 50k genotypes will therefore require training individuals from every target breed, confirming findings from simulations [48].

Recently released next generation Illumina HD or Affymetrix Bos-1 panels, with more than a 10-fold increase in SNP density beyond the 50k, will allow imputation of missing SNP genotypes in animals already genotyped for 50k panels [45,46]. It is hoped that the 10-fold increased SNP density will improve across-breed prediction, avoiding the need for large training populations of every target breed, but this has yet to be demonstrated in practice.

Genomic prediction across-breed using reduced panels will be inferior to 50k based predictions. A subset of 192 SNP markers was chosen from the US-MARC association analysis for weaning weight reported in [38] and applied to predict merit for weaning weight and post-weaning gain in purebred calves representing seven of the breeds represented as crossbreds in the US-MARC training data. The genetic correlation estimated between the MBV and direct effects for weaning weight was slightly negative (-0.05) in one breed, 0.0 in another, and ranged from 0.10-0.28 in the remaining breeds [39]. These results are disappointingly low.

### Incorporation of genomic information in US national cattle evaluation

Both predictions from Merial Igenity and Pfizer Animal Genetics are currently used in the American Angus Association (AAA) NCE by including them as correlated traits. The estimated genetic correlations for the Merial Igenity MBV are 0.54 for carcass weight, 0.58 for REA, 0.50 for fat and 0.65 for marbling [25]. Corresponding values have not yet been reported for the Pfizer Animal Genetics MBV. Procedurally, breeders send DNA samples to AAA, where they are anonymously recoded and forwarded to the relevant genomics company. The MBV are reported back to AAA to be provided to the breeders and included in NCE. In this circumstance, retraining to improve the accuracy of genomic prediction is not an option as no party has access to both the genotypes and EPD or phenotypic performance of the genotyped individuals.

### Future hopes

Predictive ability is influenced by effective population size, heritability, and the number of animals in the training data, among other factors [20,29]. Increasing the number of genotyped animals should increase predictive ability. Ideally, the training data should accumulate as the seedstock producers genotype individuals for selection purposes. Unlike for the dairy industry, this is not occurring yet in the beef industry, since genomics companies are marketing predictions without the genotypes going into the national databases administered by the breed associations. Research populations may therefore be critical to the accumulation of training animals in the near term. In Australia, industry has actively promoted an information nucleus for this very purpose [49]. The presence of such populations will inevitably place strain on the relationship between genomics companies that want to keep information of a proprietary nature and public/industry funding efforts. Pooling training populations across countries provides an opportunity to increase training data size, but may add complications. Different countries sometimes define traits in different ways (e.g. age-adjusted or weight-adjusted), and have different harvest end-points (e.g. weight-constant or fat-constant), resulting in imperfect relationships between the traits in different countries. Further, genotype by environment interactions can also be important because production conditions tend to be more diverse in beef cattle than in dairy production. Pooling training data across breeds provides an appealing alternative to increase predictive power but will require the use or imputation of new higher-density SNP panels. The use of haplotypes [50] may also provide additional power, although this has yet to be demonstrated in beef cattle with field rather than simulated data. Cost-effective use of the technology will likely result in approaches that exploit genotype imputation, and use mixed densities of genotyping on individual animals. This will likely include the DNA sequencing of individual animals [51], such as widely-used AI sires, and the imputation of sequences. However, additional SNP information alone may reduce predictive ability [47] unless the size of the training populations increases. Exploiting bioinformatics, such as from expression analyses and knowledge of the location of genes known to influence traits in beef cattle or other species, may help to increase predictive ability by allowing focusing on additional SNP only in the regions that lack sufficient LD. New analytical methods, such as approaches that explicitly fit QTL effects [52] rather than SNP effects (such as methods that jointly account for LD and linkage information [53]) may also help.

Extension of genomic predictions to the full range of traits that influence consumer satisfaction will further require a focus on the collection of reliable phenotypic information across the broad spectrum of traits. Collecting such information will likely rely on public funding efforts, but even then will be limited by the availability of meaningful phenotypes for some traits. New electronic technologies that facilitate the collection of phenotypes on large cohorts will also be invaluable.

## Conclusion

Genomic prediction offers accuracies that exceed those of pedigree-based parent average of young selection candidates. The highest accuracies are achieved for offspring of the training population. Accuracies can be equivalent to progeny tests based on up to 10 or so offspring, providing a slightly higher predictive ability than a single phenotypic observation on the individual. These accuracies are not yet sufficiently high to warrant selection in the absence of phenotypic information, particularly as these accuracies tend to erode when assessed in validation populations that are more distant from the training population in terms of the number of meioses separating generations. Accuracies are expected to improve with further research, as the training population grows in terms of numbers of genotyped animals, and density of SNP genotypes per animal.

Phenotyping is now the principal limitation in expanding the series of traits beyond those routinely recorded for NCE. In the meantime, applying genomic prediction will influence traits that were easy to record in conventional improvement programs, rather than addressing the traits difficult and costly to measure.

Sharing of information among parties to the benefit of industry is still in its infancy, as is the incorporation of MBV into NCE. The latter activity will cause particular challenges for small breed associations which lack the funding or expertise to change their NCE systems. Whereas it had been hoped that genomic prediction would facilitate selection in small breed associations with fewer registered animals, the current need for within-breed training will serve only to increase the technology gap between the breeds and facilitate faster rates of change in those breeds that have a large market share.

## List of abbreviations used

CMP: (carcass merit project); EBV: (estimated breeding value); EPD: (expected progeny difference); LD: (linkage disequilibrium); IMF: (intramuscular fat); MBV: (molecular breeding value); NBCEC: (National Beef Cattle Evaluation Consortium); NCBA: (National Cattlemen's Beef Association); NCE: (national cattle evaluation); PA: (parent average); QTL: (quantitative trait locus); REA: (rib-eye area); SNP: (single nucleotide polymorphism); US-MARC: (United States Meat Animal Research Center).

## Competing interests

The author declares that they have no competing interests.
